# An ultrasound study of altered hydration behaviour of proteoglycan-degraded articular cartilage

**DOI:** 10.1186/1471-2474-14-289

**Published:** 2013-10-11

**Authors:** Qing Wang, Yi-Yi Yang, Hai-Jun Niu, Wen-Jing Zhang, Qian-Jin Feng, Wu-Fan Chen

**Affiliations:** 1 Institute of Medical Information, School of Biomedical Engineering, Southern Medical University, Guangzhou 510515, China; 2 Key Laboratory of the Ministry of Education for Biomechanics and Mechanobiology, School of Biological Science and Medical Engineering, Beihang University, Beijing 100191, China

**Keywords:** Hydration, Proteoglycans, Articular cartilage, Ultrasound, Osteoarthritis

## Abstract

**Background:**

Articular cartilage is a solid-fluid biphasic material covering the bony ends of articulating joints. Hydration of articular cartilage is important to joint lubrication and weight-wearing. The aims of this study are to measure the altered hydration behaviour of the proteoglycan-degraded articular cartilage using high-frequency ultrasound and then to investigate the effect of proteoglycan (PG) degradation on cartilage hydration.

**Methods:**

Twelve porcine patellae with smooth cartilage surface were prepared and evenly divided into two groups: normal group without any enzyme treatment and trypsin group treated with 0.25% trypsin solution for 4 h to digest PG in the tissue. After 40-minute exposure to air at room temperature, the specimens were immerged into the physiological saline solution. The dehydration induced hydration behaviour of the specimen was monitored by the high-frequency (25 MHz) ultrasound pulser/receiver (P/R) system. Dynamic strain and equilibrium strain were extracted to quantitatively evaluate the hydration behaviour of the dehydrated cartilage tissues.

**Results:**

The hydration progress of the dehydrated cartilage tissue was observed in M-mode ultrasound image indicating that the hydration behaviour of the PG-degraded specimens decreased. The percentage value of the equilibrium strain (1.84 ± 0.21%) of the PG-degraded cartilage significantly (p < 0.01) decreased in comparison with healthy cartilage (3.46 ± 0.49%). The histological sections demonstrated that almost PG content in the entire cartilage layer was digested by trypsin.

**Conclusion:**

Using high-frequency ultrasound, this study found a reduction in the hydration behaviour of the PG-degraded cartilage. The results indicated that the degradation of PG decreased the hydration capability of the dehydrated tissue. This study may provide useful information for further study on changes in the biomechanical property of articular cartilage in osteoarthritis.

## Background

Articular cartilage is a hydrated-charged connective tissue that covers the bony ends in articulating joints, providing a smooth efficient weight-bearing system for the body. Previous studies have discovered that articular cartilage can be described as a solid-fluid biphasic mixture. Proteoglycan (PG), collagen fibril, and water are the three major components of articular cartilage [1,2]. PG as an important component has a complex chemical structure [3]: one or more glycosaminoglycans (GAGs) are attached to a protein core forming aggrecan and aggrecans bind to hyaluronan (HA) composed of large PG aggregates forming PG macromolecule. The noncovalent bond in the charged sulphate and carboxyl groups along PG could attract water. Proteoglycans (PGs) and collagen fibrils are composed of a porous matrix (collagen-proteoglycan extra-cellular matrix, collagen-PG ECM), which is the solid phase. The fluid phase includes the interstitial water and the dissolved ions. Water is the most abundant component of articular cartilage making the collagen-PG matrix swollen and being a cohesive and stiff matrix. The water content reflects the degree of hydration of articular cartilage.

Two types of water exist in articular cartilage, i.e. free water and bound water. The former depends on osmosis pressure to permeate in or shift out of the cartilage tissue. The later is related to the PGs and collagen network. Therefore, the amount and the spatial distribution of the interstitial water in the cartilage layer are mainly related to the PG concentration and the orientation of collagen network [2]. The interaction between collagen network, PG and water is believed to play an important role in the biomaterial and biomechanical properties of articular cartilage [2,3].

In natural osteoarthritis (OA), several factors are involved in the progress of the PG degeneration. First, the abnormal loading on the cartilage matrix can cause quick failure of articular cartilage. The high magnitude of the imposed stresses and the sustained stress peaks may trigger an imbalance of chonchrocyte anabolic and catabolic activities, leading to the abnormal metabolism of PG. Without the normal function of chonchrocytes, the decomposition of PG is more than the composition of PG, thus the cartilage matrix greatly loses the PGs [4]. Secondly, some enzymes such as metalloproteinase including trypsin and aggrecanase digest the PGs in the cartilage matrix causing the degeneration of PG [5]. Thirdly, the structural damage of the PGs and collagen fibrils triggers immunological reaction and consequently the degradation of PG is accelerated [6].

Hydration after dehydration is very important for articular cartilage to remain its normal function. A previous study found that the surface layer of the dehydrated cartilage tends to be more easily ruptured than the hydrated cartilage [7]. However, the ability of the PG-degenerated cartilage to recover its hydration after dehydration has not been investigated. Previous studies found that the increased water content in early OA might be related to the change of collagen network as well as the breakdown of PG architecture [8], which results in an increase in permeability of the solid matrix and consequently changes hydration of the matrix [9,10]. Furthermore, the reduction in the hydration level resulted in an increase in the modulus of the cartilage tissue under compression [11].

To study the hydration of articular cartilage, the degree of cartilage hydration was evaluated by the measurement of water content using water weighting or freeze-drying methods, while the progress of cartilage hydration could be probed using ultrasound [12,13]. However, the detail explication was lack for the hydration behaviour of articular cartilage in those studies. The effect of the degradation of the PGs on hydration of the dehydrated cartilage tissue has not been clearly investigated, although the key role of the negatively changed PGs in the osmosis-induced swelling of articular cartilage has been reported [9,14]. Therefore, the function of PG in the dehydration-induced hydration behaviour of articular cartilage would be investigated in this study.

Previous studies have indicated the potentials of ultrasound technique in quantitative evaluation of the transient behaviour of normal and degenerated articular cartilage [15,16]. This study therefore applied high-frequency ultrasound to monitor the hydration progress of the dehydrated cartilage. Selectively targeting the PGs in the matrix, this study applied the trypsin enzyme to digest the PGs in the cartilage samples. The aims of this study are to monitor the altered hydration behaviour of the PG-degraded articular cartilage and to explore the role of PGs in water intake of the dehydrated cartilage tissue.

## Methods

### Preparation of specimens

Twelve porcine patellae without obvious lesions were obtained from the local slaughterhouse and evenly divided into two groups. Normal group (n=6) was treated as control group without any enzyme digestion. The specimens in trypsin group (n=6) were submerged in 0.25% trypsin solution (Trypsin, JingKeHongDa Bio-tech Co., Ltd., Beijing, China) to digest the PGs in the tissue. After 4-hour trypsin treatment, the trypsin solution was removed and the PG-degraded specimens were washed with PBS solution for three times. Experiments on porcine patellae were approved by our institutional animal care and use committee and performed under the guidelines of the National Institutes of Health for the care of laboratory animals.

All of the specimens were frozen at -20°C for the hydration test by ultrasound examination. On the day of testing, the cartilage specimen was removed from the freezer and thawed for one hour in the physiological saline at room temperature.

### Experimental system

**Figure 1:**
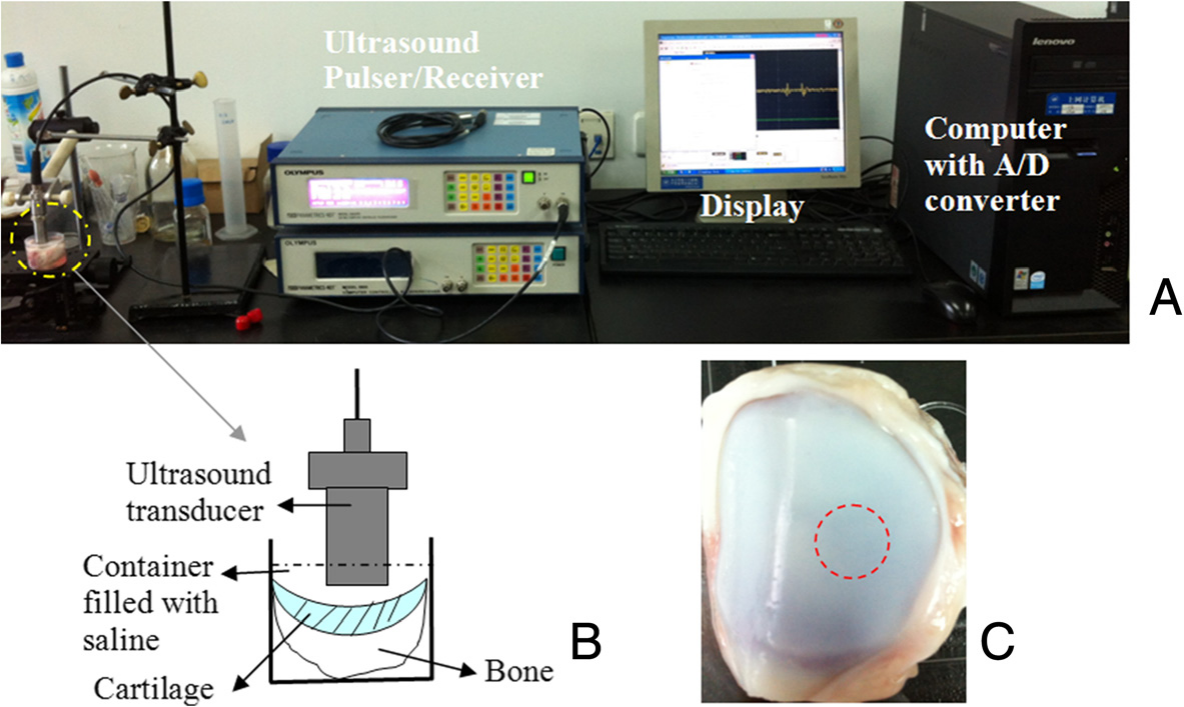
**Ultrasound pulser/receiver (P/R) system used to scan porcine patellae. (A)** Experiment setup of the system including computer with A/D card and signal processing software, computer display, ultrasound pulser/receiver, container filled with the saline solution, and ultrasound transducer. **(B)** An enlarged view of the essential components in dashed circle in **(A)**. **(C)** A typical sample of porcine patella. The dashed circle indicates the site scanned by ultrasound.

Figure 1A shows the experiment setup. The ultrasound measurement system includes an ultrasound pulser/receiver (OLYMPUS 5900PR, Panametrics-NDT, USA), a 12-bit A/D converter card with a sampling rate of 200 MHz (CS12400, Gage, Canada), and a Lenovo computer. A 25 MHz ultrasound transducer (OLYMPUS Panametrics-NDT V356, USA) was used to transmit ultrasound pulses into the tissue via the physiological saline solution and to receive the ultrasound echoes. Ultrasound pulser/receiver (P/R) was applied to drive the ultrasound transducer and to amplify the received ultrasound echoes. Pulse repetition frequency (PRF) of the ultrasound P/R system was set at 200 Hz. A/D converter board triggered by the transmitting trigger of the ultrasound pulser/receiver was used to digitize the ultrasonic signals. The A-mode ultrasonic signals were displayed and recorded with the GageScope software (ver. 3.80.02, Gage, Canada).

The central portion of the medial part of the patellar was selected as the ultrasound-monitored site (Figure 1C). As shown in Figure 1B, the patellar was installed and fixed in a small cup and submerged in the saline solution. The ultrasound transducer was located perpendicularly above the cartilage surface with aligning the ultrasound beam into the cartilage tissue to obtain the maximum echo amplitude.

### Dehydration-induced hydration test

Before the hydration test, the patellar specimen was equilibrated in physiological saline. The transducer was located over the cartilage surface as shown in Figure 1B. The echoes from the cartilage could be clearly observed on the display of the computer.

Then, the saline solution was removed with an injector and a piece of paper tissue was used to gently sip the residual water on the surface. The cartilage surface was exposed to the air at room temperature for 40 minutes. After 40-minute dehydration, the physiological saline was refilled to submerge the cartilage surface. As the echoes from the tissue appear on the display of the computer again, the ultrasound P/R system started to record the signals at a sampling rate of 200MS/s. The hydration behaviour was monitored for 40 minutes by the system. The transducer and the specimen were not moved during the entire procedure of the experiment. All the tests were performed at a room temperature of 25°C±1°C and humidity of 65% ± 5%.

### Hydration strain calculation

In this study, a cross-correlation echo tracking method [17,18] was used to track the shift of the selected ultrasound echoes from the superficial layer so as to measure the corresponding time of flight (*∆T*), which could be used to calculate the deformation (*∆h*) of the cartilage layer according to equation (1):

(1) $$\Delta h=v_{s}\Delta T/2$$

where *v*_*s *_is the mean sound speed in physiological saline and equal to 1532 m/s [19]. The thickness (*h*_*0*_) of the cartilage tissue in physiological saline was calculated with the mean sound speed in cartilage (*v*_*c*_) and the time of flight (*T*) in the tissue by Equation (2).

(2) $$h_{0}=v_{c}T/2$$

where *v*_*c *_equal to 1675 m/s [19]. Then the hydration strain *ϵ* was calculated using the following equation:

(3) $$\epsilon =\frac{\mathrm{\Delta h}}{h_{0}}$$

### Histological assessment

After ultrasound examination, the ultrasound-scanned part (approximately 1cm × 1cm with 4-mm thickness) was excised from each patella. Then the samples were processed according to the standard protocol of tissue processing. The samples were fixed in a 10% buffered formalin and then decalcified in a 10% ethylenediamine tetraacetic acid (EDTA) solution. Paraffin sections of 4-μm thickness were prepared near the central portion of the sample. The deparaffined sections were stained with Safranin O (Cart. No. 2062C038, amresco, USA) and contrastained with fast green (Cat. No. 201010, Qiyun Bio-tech Co., Ltd., Guangzhou, China). In the optical micrographs, red color due to safranin O indicates the presence of PG. The PG-degradation zone was not stained with safranin O.

### Statistical analysis

Statistical analyses were conducted with SPSS software (V17, SPSS Inc., Chicago, IL, USA). All values in the text are presented as mean ± SD. In light of the small number of samples, nonparametric analysis was used in this study. The Mann-Whitney U test was used to test the statistical difference in the equilibrium hydration between normal cartilage specimens and the PG-degraded specimens. Statistical significance was considered at p < 0.05.

## Results

### Hydration behavior of articular cartilage

**Figure 2:**
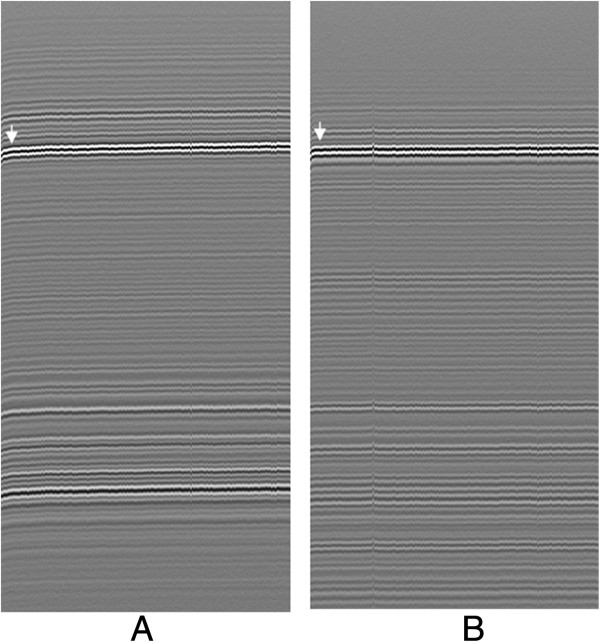
**Hydration behaviour of articular cartilage induced by dehydration displayed in the M-mode ultrasound image of a representative normal sample (A) and a representative PG-degenerated sample (B).** White arrows indicate the deformation of the cartilage surface during hydration process.

The response of the dehydrated cartilage tissue after re-submerged in physiological saline was represented in M-mode ultrasound image (Figure 2). It is indicated that the cartilage tissue was swollen and hydrated rapidly. We tracked the deformation of the cartilage surface and observed that the hydration amplitude of the PG-degraded specimens decreased in comparison with the normal ones.

### Biomechanical measurement

**Figure 3:**
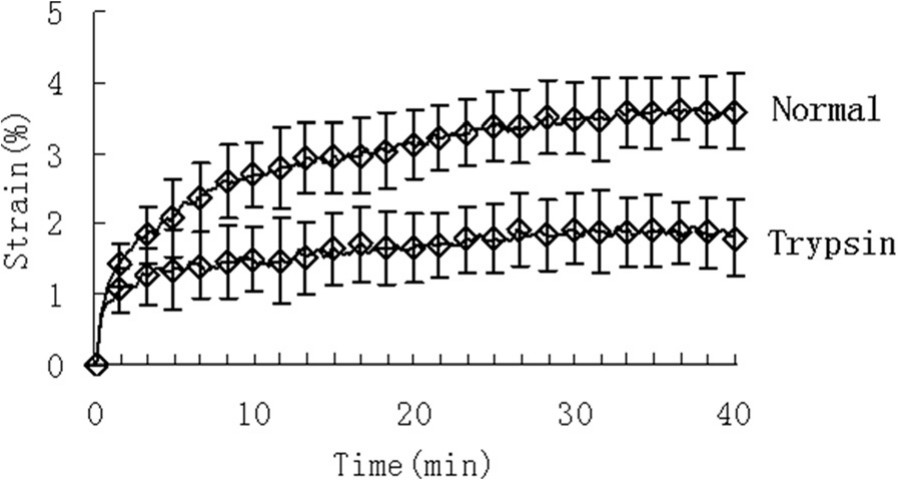
Dynamic strain increased in a creep curve both for normal group and Trypsin group.

**Figure 4:**
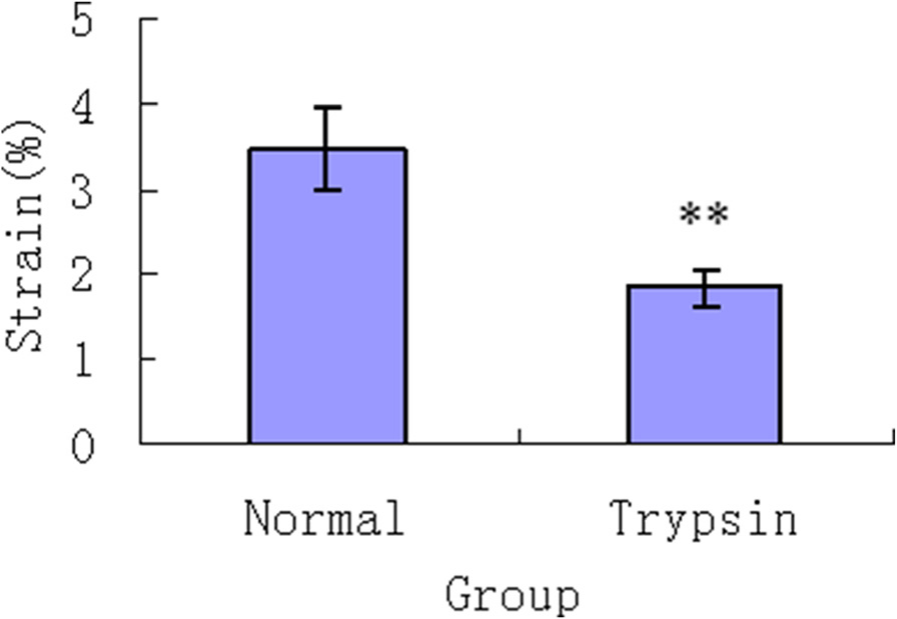
**Equilibrium strain of normal group and Trypsin group.** ** denotes significant difference at p < 0.01.

Figure 3 shows that the dynamic strain increased in a creep curve both for normal group and trypsin group. But the dynamic strain of normal group reached the equilibrium within approximately 20 minutes, whereas the PG-degraded specimens quickly balanced within averagely 5 minutes. Additionally, as shown in Figure 4, the percentage value of the equilibrium strain (1.84 ± 0.21 %) of the PG-degraded cartilage significantly (p < 0.01) decreased in comparison with healthy cartilage (3.46 ± 0.49 %).

### Histological analysis

**Figure 5:**
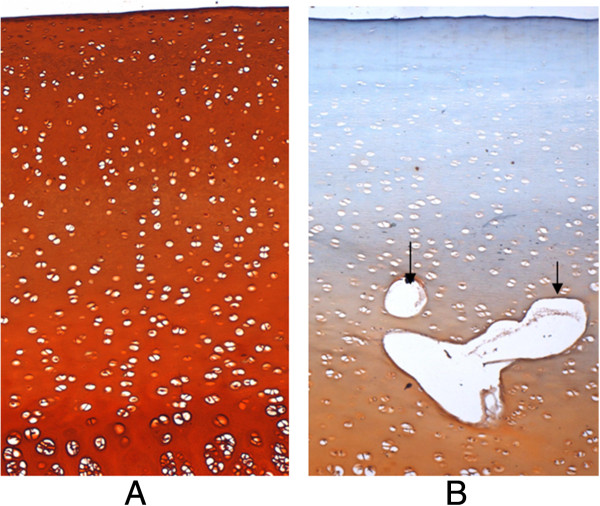
**Histological sections stained with Safranin O and fast green. (A)** Histological section of normal cartilage indicating smooth surface, the well-arranged cartilage cells in a rule, no erosion, ulcers or fissures in the cartilage tissue, and the uniform staining with Safranin O red. **(B)** Histological section of PG-degraded cartilage indicating less smooth surface in comparison with normal cartilage in **(A)**, the destructed local chondrocytes in the cartilage forming a cavity (arrows), moderate reduction of Safranin O staining indicating a degeneration of proteoglycans in the cartilage matrix. (× 100).

The histological images in Figure 5 clearly indicated that trypsin treatment digested the PGs in the cartilage tissue. Under light microscopy, smooth surface, the well-arranged cartilage cells in a rule, no erosion, ulcers or fissures in the cartilage tissue was found in normal articular cartilage, which was uniformly stained in red colour with safranin O (Figure 5A). No severe surface irregularity or cleft was found in the PG-degraded articular cartilage (Figure 5B). It however was found that the safranin O staining reduced in the interterrritorial matrix, indicating the degeneration of the PGs in the cartilage matrix. Additionally, the destructed local chondrocytes formed cavities in the cartilage tissue.

## Discussion

### PG and water in health and enzyme-treated articular cartilage

In the biphasic cartilage, the fluid phase plays an important role in regulating the structural organization of the ECM, the swelling properties of articular cartilage, and joint lubrication [3]. Most of water occupies the interfibrillar space of the ECM and is free to move under a load or pressure gradient or other electrochemical motive forces applied to the tissue [20]. A small amount of water is bound by the PGs and collagen fibrils. The amount of bound water decreases due to the loss of noncovalent bond along PG. However, on the other hand, because the PGs are decomposed, the porosity and permeability of the matrix resultantly increase [9,10]. The long chains of normally aggregated macromolecular PGs are broken into smaller sections [1], which become unstable and are easily released from the matrix [3]. The degree of water content of the PG-degenerated cartilage samples emerged in physiological saline might increase in comparison with the normal ones. It has been well known that water content increases in the OA cartilage with the degeneration of PGs and the damage of collagen network [21-23].

In this study, the loss of water was induced by a method of exposure-to-air and then the hydration behaviour of articular cartilage re-submerged in physiological saline was monitored by ultrasound. Our finding that the magnitude of hydration behaviour reduced for the PG-degenerated cartilage is similar to a previous study [2]. It could be explained by the following two possible reasons. On one side, the PG-degenerated tissue lost greater amount of water than the normal one during dehydration. On the other side, the lower osmotic stress induced less absorption of water during hydration progress. Therefore, the loss of the PGs results in the reduced water intake and retention of the dehydrated matrix in comparison with healthy dehydrated cartilage tissue.

In natural OA, changes in water content of articular cartilage usually are associated with composition and structural alterations of the collagen-PG ECM as well as synovial fluid [3]. Some studies found that GAG loss might not be only one of the effect factors of changing cartilage hydration [24,25]. To investigate the alteration in hydration behaviour of the PG-degraded dehydrated cartilage, this study employed enzymatic digestion, which is a well-known method to simulate OA-like cartilage in experimental study and has been widely utilized to selectively target one or more crucial components of cartilage, such as collagen and/or PG. We used trypsin to degrade the PGs. Besides the decomposition of PG macromolecules, the trypsin treatment causes minor degeneration of the collagen network [26]. The minor damage in collagen network especially in the superficial layer might increase the evaporation of water during dehydration. However, the effect of minor damage in collagen network on the osmotic stress in the cartilage tissue is not as great as the PGs, because the changes in the collagen network could not be qualitatively measured in polarized light microscopy images [27]. In addition, we found that the trypsin treatment might destruct the local chondrocytes to formed the cavities (Figure 5B). However, most PGs were degenerated by the enzyme and thus the effect of the destructed chondrocytes on PG synthesis was slight.

### Hydration behaviour compared with osmotic-induced swelling behaviour

This study employed exposure-to-air to obtain the dehydrated cartilage tissue. After dehydration, the increase of the ion concentration in the tissue resulted in an increase of swelling stress. When re-submerged in physiological saline, the tissue gains water and becomes swollen. This phenomenon is called hydration behaviour of articular cartilage. During hydration progress, free water played a dominant role while the movement of bound water was limited by the PGs and collagen network. Moreover, fewer ions were involved in hydration progress, because cations are attracted by the negative charge groups on the GAGs [3]. These may explain why the dehydration-induced hydration of articular cartilage behaves in gradually reaching to equilibrium.

Unlike hydration behaviour, the osmosis-induced swelling of articular cartilage behaves in an “overshoot-relaxation” way [15]. The osmosis-induced swelling of articular cartilage was closely related to water content and ion diffusion under the Donnan osmotic pressure [9,28]. Because of the change in the concentration of the bathing saline from 2 mole/L to 0.15 mole/L, the Donnan osmotic pressure made the tissue gained water and swollen [15]. Possibly due to the re-distribution of ions along the depth of the tissue, the cartilage tissue showed a peak of swelling and then slowly reached to equilibrium [15]. The ions play an important role in cartilage swelling and thus are considered as the third phase in the triphasic cartilage [9]. This difference between hydration behaviour and swelling behaviour might be resulted from the loss of water in the cartilage layer caused by the vapour pressures rather than the ion transmission induced by changing the ion concentration of the bathing solution. The results of this study and previous studies indicate the importance of the PGs in both cartilage hydration and cartilage swelling.

With the PGs, the dehydrated healthy cartilage tissue could recovery the hydration when re-submerged in physiological saline. The fixed negative charge groups on the GAGs that attract cations are greatly important for water retention of the matrix [2,3]. The interaction and association of PG, ions and water influence the mechanical properties of articular cartilage. Recently, it has been reported that the dehydrated cartilage is easily damaged due to less available fluid available for stress sharing [7]. As mentioned above, after the macromolecular PGs were degraded, the hydration of articular cartilage decreased with the loss of hydrophilic function of the PGs.

### Ultrasound quantitative measurement

The approach of high-frequency ultrasound has been used to measure the osmosis-induced swelling behaviour of healthy and degraded articular cartilage [14-16,29]. Considering the high resolution, we applied the 25 MHz ultrasound system to monitor the hydration behavior of the dehydrated cartilage tissue and then tracked the deformation of the tissue to obtain the dynamic strain and the equilibrium strain for a quantitative evaluation of cartilage hydration. Real-time monitoring and quantitative measurement are the advantages of the ultrasound approach.

In previous studies, the cartilage tissue had to be excised from the subchondral bone for calculation of water content. The hydration of the cartilage tissue was assessed by freeze-drying for approximately 24 hours and then the water content is calculated as the difference between wet and dry weight [30]. It means the freeze-drying method can hardly complete the *in vivo* or *in situ* test. Additionally, the freeze-drying approach is not suitable for assessment of the capability of the dehydrated tissue to hydrate with water. The *in-situ* cartilage-on-bone condition of the cartilage tissue may be one of important effect factors for studies on the mechanical properties of articular cartilage [30-32]. Therefore, the ultrasound measurement system provides a non-contact and non-destructive approach to assess the mechanical properties of articular cartilage indicating its advantage in *in-vivo* or *in-situ* measurements. In this study, we measured the hydration of articular cartilage at the central region of the intact patella *in situ* without excision of the surround cartilage and bone tissues.

## Conclusion

In summary, this study demonstrated the changes in hydration of the PG-degraded cartilage. 40-minute dehydration induced water loss in articular cartilage. Real-time M-mode ultrasound imaging indicated that the dehydrated cartilage gradually swelled to equilibrium without “overshoot-relaxation” of the osmosis-induced swelling. The results of this study suggested that the hydration capability of the dehydrated PG-degenerated cartilage decreased significantly in comparison with healthy cartilage. The reported ultrasound method could be potentially used for the *in situ* measurement of hydration of intact cartilage layer.

## Competing interests

All authors have no competing interests according to the products used.

## Authors’ contributions

QW, YYY, HJN, and WJZ were involved in the design of the study, acquisition of data, and analysis of data. QW, QJF, and WFC were responsible for drafting the paper and revising it. And all authors commented on the draft. All authors have read the manuscript and have given final approval of the version to be published.

## Pre-publication history

The pre-publication history for this paper can be accessed here:

http://www.biomedcentral.com/1471-2474/14/289/prepub
